# Measuring Solvation Interactions of Deep Eutectic Solvents Formed by Metal Chlorides and an Imidazolium Salt by Inverse Gas Chromatography

**DOI:** 10.1002/jssc.70231

**Published:** 2025-08-05

**Authors:** Siyuan Liu, Jared L. Anderson

**Affiliations:** ^1^ Ames National Laboratory U.S. Department of Energy Ames Iowa USA; ^2^ Department of Chemistry Iowa State University Ames Iowa USA

**Keywords:** deep eutectic solvent, gas chromatography, inverse gas chromatography, solvation interactions, stationary phase

## Abstract

Deep eutectic solvents (DESs) represent a class of solvents that offer a number of advantages including minimal toxicity, affordability, low vapor pressure, and simple, environmentally friendly preparation methods. They have found utility in areas, such as gas absorption, metal plating, and extractions. However, the relationship between their solvation properties and chemical composition remains poorly understood. In this study, a broad range of Type I DESs composed of metal chlorides and an imidazolium salt were prepared, employed as gas chromatographic stationary phases, and characterized using the Abraham solvation parameter model by inverse gas chromatography. The Abraham solvation parameter model allows for the study of DES solvation properties and the effects of varying structural components on system constants using a linear‐free energy relationship. The DESs were investigated by systematically varying their composition, including the type of metal chloride and the molar ratio between the metal chloride and the imidazolium salt. The results show that hydrogen bond acidity, hydrogen bond basicity, and dipolarity/polarizability interactions are strongly influenced by the type of metal chloride within the DES and the ratio of metal chloride to imidazolium salt in the eutectic mixture. Furthermore, a column pretreatment procedure is presented that enables the effective coating of highly polar DESs onto open tubular capillary gas chromatography columns.

## 1 Introduction

Deep eutectic solvents (DESs) are a class of solvents that are liquids at or near room temperature [1] and have gained considerable attention due to their customizability and environmental friendliness [2, 3]. A eutectic mixture often includes a hydrogen bond donor (HBD) and a hydrogen bond acceptor (HBA) and possesses melting points lower than that of the individual constituents [4]. Depending on the HBA and HBD composition, they can be biodegradable [5] and involve low‐cost and easy preparation procedures, while possessing low melting temperatures [6], minimal vapor pressures [7], and tunable viscosities [8]. Due to their varied physicochemical properties, DESs have been employed in a number of applications within the field of chemistry, including metal electrodeposition [9], carbon dioxide absorption [10], desulfurization of fuels [11], and biomass delignification [12]. Based on their HBA/HBD constituents, the majority of DESs that have been documented to date can be divided into the following primary categories [13]: Type I, comprised of organic salts and metal chlorides; Type II, containing organic salts and metal chlorides hydrates; Type III, comprised of organic salts and HBDs; and Type IV, containing metal chlorides and HBDs. An understanding of DES solvation characteristics is important in describing the interactions that solutes have with DESs, as this is important in optimizing their use as solvents in chemical reactions [13], chemical separations [14], biological assays [15], drug delivery [16], and material science [17]. The individual solvation interactions in DESs that are important include hydrogen bond acidity, hydrogen bond basicity, dipolarity/polarizability, and dispersive‐type interactions [18].

Type I DESs comprise a wide range of chlorometallate ionic solvents that were widely studied in the 1980s [19], and include imidazolium chloroaluminates which are based on mixtures of aluminum trichloride (AlCl_3_) and 1‐ethyl‐3‐methylimidazolium chloride ([EMIM^+^] [Cl^−^]) [19]. Depending on the employed concentration of AlCl_3_, chloroaluminate electrolytes produced by mixing AlCl_3_ and [EMIM^+^] [Cl^−^] can be classified as basic, neutral, or acidic using Raman spectroscopy [20]. In particular, electrolytes consisting of [AlCl_4_^−^] and [Cl^−^] ions have been shown to exhibit basic characteristics when the AlCl_3_: [EMIM^+^] [Cl^−^] molar ratio is lower than 1.0 [20]. On the other hand, the electrolyte becomes acidic when the AlCl_3_: [EMIM^+^] [Cl^−^] molar ratio is higher than 1.0, as they are comprised of [AlCl_4_^−^] and [Al_2_Cl_7_^−^] species [20]. It has been reported that only the acidic DES composition exhibits activity for aluminum plating and stripping at the anode, as indicated by the following reversible reaction: 4Al_2_Cl_7_^−^ + 3e^−^ ↔ Al + 7AlCl_4_^−^ [20]. Conversely, the basic DES composition has demonstrated poor electrochemical activity [20]. Clearly, some Type I DESs with certain HBA/HBD ratios are more better suited than others in applications like aluminum plating, and a more comprehensive working knowledge of their solvation capacities is required to enhance and eventually predict their performance.

Raman spectroscopy has been used to investigate the AlCl_3_‐[EMIM^+^] [Cl^−^] system, and the intensities of the four peaks at 98, 310, 157, 428 cm^−1^ have been observed to increase when a larger AlCl_3_ concentration is present within the DES, with these peaks being assigned to the [Al_2_Cl_7_^−^] ion. It was proposed that the intensity of these peaks could be used to predict the acidity/basicity of the AlCl_3_‐[EMIM^+^] [Cl^−^] system [21]. Nevertheless, Raman spectra do not readily provide a quantitative measurement of the acidity and basicity of type one DESs formed by metal chlorides and imidazolium salts. To more deeply understand the complex solvation characteristics of these Type 1 DESs, it is crucial to consider not only their acidity and basicity but also other solvation properties, such as *n*–π and π–π interactions, dipolarity/polarizability, and dispersive interactions, which are not easily accessible through Raman spectra.

Inverse gas chromatography (IGC) is an approach that complements traditional analytical GC and is primarily used to characterize the surface and bulk properties of a stationary phase [22, 23]. While analytical GC separates mixtures of volatile compounds into their individual components [24], IGC involves an examination of the chromatographic retention characteristics of known probe molecules to stationary phase materials [25]. When IGC is used to investigate the solvent properties of a particular stationary phase [26], the liquid‐polymer stationary phase is coated on the wall of an open tubular GC column [27] or coated or immobilized as a stationary phase on a solid support [28]. A range of probe molecules are used to interrogate the solute–solvent interactions of the stationary phase by measuring the chromatographic retention of specific probe molecules [23]. IGC has previously been employed to investigate the solvation properties of Type 3 DESs, which are formed upon mixing phosphonium/ammonium salts and HBDs like amides, carboxylic acids, or alcohols [29, 30, 31]. Until now, no studies have investigated the solvation properties of the other three types of DESs.

In this study, IGC is employed to measure the multiple solvation interactions of four type I DES systems under varying temperatures, each comprised of the 1‐butyl‐3‐methylimidazolium chloride ([BMIM^+^] [Cl^−^]) as HBA and ferrous chloride (FeCl_2_), ferric chloride (FeCl_3_), zinc chloride (ZnCl_2_), and AlCl_3_ as HBD. When metal‐containing DESs are applied as stationary phases on untreated capillary columns to fabricate wall‐coated open tubular columns, their high polarity leads to droplet formation of the stationary phase. This phenomenon results in poorly coated columns with low chromatographic efficiency, rendering them unsuitable for IGC characterization. To increase the glass surface's wettability, a process for roughening the surface of GC columns using suspensions of sodium chloride and silicon dioxide is presented to achieve better wetting of the stationary phase on the column wall and facilitate the measurement of highly precise chromatographic retention times and separation efficiencies. It was found in this study that variations in the metal chloride within the eutectic mixture resulted in the greatest change in hydrogen bond acidity. Eutectic mixtures possessed the highest hydrogen bond acidity when FeCl_3_ was used as HBD. Different HBA/HBD ratios within each DES system were examined and it was found that even when the HBA/HBD ratio is varied in 0.5 molar increments, the solvation interactions of eutectic mixtures, particularly the hydrogen bond acidity, hydrogen bond basicity, and dipolarity/polarizability are significantly influenced and can be effectively modulated.

## 2 Experimental

### 2.1 Reagents and Materials

The reagents 1‐chlorobutane (99%), cyclopentanol (99%), methyl caproate (99%), naphthalene (99%), ethyl acetate (99.5%), nitromethane (99%), ferrous chloride (98%), and 1‐methylimidazole (99%) were purchased from Acros Organics (Morris Plains, NJ, USA). Ethyl benzene was purchased from Eastman Kodak Company (Rochester, NJ, USA). Chloroform (99.8%), toluene (99.8%), and 1‐hexanol (98%) were purchased from Fisher Scientific (Pittsburgh, PA, USA). Methyl acetate (98%), *o*‐xylene (97%), *p*‐xylene (99.5%), and 1‐bromohexane (98%) were purchased from Fluka (Steinheim, Germany). Benzaldehyde (99%), 1‐chlorooctane (99%), cyclohexanol (99%), 1‐chlorohexane (99%), cyclohexanone (99.8%), 1‐nitropropane (98%), octylaldehyde (99%), 1‐pentanol (99%), propionitrile (99%), 2‐pentanone (99%), benzonitrile (99%), acetophenone (99%), 2‐butanol (98%), 1‐butanol (99.8%), 1‐bromooctane (99%), 1,2‐dichlorobenzene (99%), dichloromethane (99.8%), 1,4‐dioxane (99.5%), *m*‐xylene (99.5%), 2‐propanol (99.9%), 1‐propanol (99.9%), methanol (99%), ethanol (99%), nitrobenzene (99%), ferric chloride (97%), zinc chloride (98%), ethoxybenzene (99%), and silica nanopowder (99.8%) were purchased from Millipore‐Sigma (St. Louis, MO, USA). Benzene (99.5%), 1‐hexyne (97%), valeraldehyde (95%), and aluminium chloride (98%) were purchased from Tokyo Chemical Industry (Portland, OR, USA). Deactivated fused silica capillary was obtained from Restek Corporation (Bellefonte, PA, USA). All reagents were used as received.

### 2.2 Preparation of DESs

The [BMIM^+^] [Cl^−^] IL was prepared by reacting equimolar amounts of 1‐methylimidazole and 1‐chlorobutane at 60°C with the mixture being heated under reflux for 4 days using a heating mantle. After cooling to room temperature, the product was washed twice with ethyl acetate. The residual ethyl acetate was evaporated by heating the product at 60°C under vacuum for 2 h using rotary evaporation [32]. Each respective metal chloride and [BMIM^+^] [Cl^−^] IL were dried in a vacuum oven at room temperature for 2 days prior to DES preparation. The DESs shown in Table 1 were prepared by directly mixing the metal chlorides and [BMIM^+^] [Cl^−^] at 70°C for 5 h until homogeneous liquids were formed [33]. Proton and carbon‐13 NMR spectra for the [BMIM^+^] [Cl^−^] IL are provided as Figures S1 and S2.

**TABLE 1:** Chemical structures, relative molar ratios of metal chloride: [BMIM^+^] [Cl^−^], and abbreviations used for the four DES systems evaluated in this study. The chromatographic efficiency of each column was measured using naphthalene at 50°C.

DES system	Metal chloride	Imidazolium salt	Metal chloride: Imidazolium salt (molar ratio)	Abbreviation of DES	Column efficiency (N/m)
1	FeCl_2_	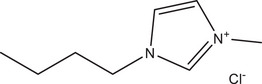 [BMIM^+^] [Cl^−^]	1:2	FeCl_2_:2 [BMIM^+^] [Cl^−^]	3183
			1:2.5	FeCl_2_:2.5 [BMIM^+^] [Cl^−^]	2253
			1:3	FeCl_2_:3 [BMIM^+^] [Cl^−^]	3314
			1:4	FeCl_2_:4 [BMIM^+^] [Cl^−^]	1911
2	FeCl_3_		1:1.5	FeCl_3_:1.5 [BMIM^+^] [Cl^−^]	2640
			1:2	FeCl_3_:2 [BMIM^+^] [Cl^−^]	2516
			1:3	FeCl_3_:3 [BMIM^+^] [Cl^−^]	2619
			1:4	FeCl_3_:4 [BMIM^+^] [Cl^−^]	2004
3	ZnCl_2_		1:2	ZnCl_2_:2 [BMIM^+^] [Cl^−^]	2087
			1:3	ZnCl_2_:3 [BMIM^+^] [Cl^−^]	1915
			1:4	ZnCl_2_:4 [BMIM^+^] [Cl^−^]	4573
4	AlCl_3_		1:4	AlCl_3_:4 [BMIM^+^] [Cl^−^]	2609
			1:5	AlCl_3_:5 [BMIM^+^] [Cl^−^]	1956

### 2.3 Pretreatment of Capillary GC Columns

The untreated column was filled with a 5 mg/mL suspension of silicon dioxide prepared by combining silica nanoparticles with chloroform and 1,4‐dioxane solvent in a 3:2 ratio and dynamically coated using a previously reported method [34]. The silicon dioxide pretreated columns were filled with a sodium chloride suspension that was prepared by mixing 8 mL of chloroform with 6 mL saturated sodium chloride methanol solution and dynamically coated by the method described by Huang et al. [35]. The columns were subjected to nitrogen flow until white crystals were formed throughout the entire column. The columns were then dried at 250°C for 20 min under nitrogen flow.

### 2.4 Preparation of Chromatographic Columns With DES Stationary Phases

Capillary columns with thin films of DES‐based stationary phases were prepared using the static coating method. Prior to coating, each DES was stored in a vacuum oven for 24 h to minimize moisture absorption. The coating solutions were prepared inside a nitrogen‐filled glove box by dissolving 44.8 mg of each DES in a solvent mixture of 9.5 mL dichloromethane and 0.5 mL methanol, resulting in a final concentration of 0.45% (w/v). Each solution was degassed for 10 min using an ultrasonic bath to eliminate dissolved gases. Pretreated 5‐m segments of fused silica GC capillary columns were then filled with the coating solution, sealed at one end, and connected to a vacuum pump at the other, with the vacuum pressure set to −10 inHg. The capillaries were subsequently placed in a water bath maintained at 42°C to promote the slow evaporation of the solvent, thereby depositing a uniform thin film of the stationary phase onto the inner wall. The film thicknesses for all DESs were approximately 0.28 µm [36]. The columns were conditioned in a gas chromatograph equipped with a flame ionization detector (FID) at 50°C for 30 min under a constant helium flow with a flow rate of 1 mL/min. Column efficiency was assessed using a naphthalene solution at the same temperature, with efficiencies ranging from 1900 to 5000 plates/m.

### 2.5 Chromatographic Separation Method

All DES stationary phases evaluated in this study were analyzed with an Agilent 7890 B (Santa Clara, CA, USA) gas chromatograph. The oven temperature was set to vary between 30°C and 50°C, while both the inlet and detector temperatures were maintained at 150°C. Helium served as the carrier gas at a flow rate of 1 mL/min, and hydrogen and air were used for operation of the FID, with flow rates of 30 and 400 mL/min, respectively. For the IGC study, all probes were dissolved in dichloromethane at a concentration of 1 mg/mL (1000 ppm), and 1 µL of each solution was injected into the GC using a split ratio of 20:1. A total of 40 probes [37] were used in the study, with retention times being measured at 30°C, 40°C, and 50°C. Propane was used to determine the dead volume of all columns at each of the three temperatures. Multiple linear regression analysis and statistical calculations were performed using Analyze‐it software (Leeds, England).

## 3 Results and Discussion

It can be expected that the type and magnitude of each individual solvation interaction for Type I DESs will differ based on the components that comprise them, particularly the respective metal chloride (HBD) and organic salt (HBA). To use Type I DESs for chemical separations, such as in fuel desulfurization, the combination of HBAs and HBDs, as well as their respective ratios, have been frequently examined to achieve the best performance. Dibenzothiophene (DBT) and thiophene are sulfur compounds found within commercial diesel that can adversely affect human health and the ecosystem [38]. Studies have shown that FeCl_3_ based DESs exhibit higher solubilities (from 17 wt% to above 90 wt%) for DBT compared to the ZnCl_2_ based DESs (0.084–1.389 wt%) [38]. Moreover, FeCl_3_ based DESs are completely miscible with thiophene while ZnCl_2_ based DESs exhibit solubility values in the range of 1−10 wt% for thiophene [38]. These findings clearly demonstrate that the chemical composition and solvation characteristics of DESs significantly influence their capacity in solvating sulfur compounds. When FeCl_3_ based DESs have been used to desulfurize fuels, the FeCl_3_: Tetra‐*n*‐butylphosphonium ([CH_3_(CH_2_)_3_]_4_PBr) (1:2) DES has been shown to exhibit higher desulfurization efficiency of DBT than the FeCl_3_: [CH_3_(CH_2_)_3_]_4_PBr (1:1.5) DES [39]. Conversely, the FeCl_3_: [CH_3_(CH_2_)_3_]_4_PBr (1:1.5) DES has shown higher desulfurization efficiency of thiophene than the FeCl_3_: [CH_3_(CH_2_)_3_]_4_PBr (1:2) DES [39]. Until now, no study has thoroughly and comprehensively measured the variation of solvation interactions for Type I DESs as the ratio of metal chloride to organic salt is systematically altered. This study addresses that gap by exploring a wide range of HBA:HBD molar ratios across four distinct Type I DES systems. By modulating the composition of these DESs, the results from this study aim to quantitate the solvation interactions with dissolved molecules to pave the way for enhanced performance when the solvents are used for targeted applications.

To relate the chromatographic retention factors for a large class of probe molecules on columns featuring stationary phases containing different DESs to determine the specific solvation interactions, the Abraham solvation parameter model was used in this work.

(1) Logk=c+eE+sS+aA+bB+lL

In Equation (1), *k* denotes the retention factors of various probes [40]. The constant term *c* elucidates the chromatographic phase ratio of the system [40]. The uppercase letters E, S, A, B, and L represent the probe solute descriptors, corresponding to the excess molar refraction, dipolarity/polarizability, hydrogen bond acidity, hydrogen bond basicity, and gas‐hexadecane partition coefficient, respectively [40]. The lowercase letters *e, s, a, b* and *l* are the system constants that characterize the specific solute‐solvent solvation interactions, including *n*–π and π–π interactions, dipolarity/polarizability, hydrogen bond basicity, hydrogen bond acidity, and dispersive‐type interactions, respectively [40].

Table 1 provides the types of metal chlorides and the chemical structure of the [BMIM^+^] [Cl^−^] IL, along with their abbreviations and corresponding molar ratios used to prepare all DESs evaluated in this study. As shown in Table 1, DES System 1 consists of ferrous chloride, with the FeCl_2_: [BMIM^+^] [Cl^−^] molar ratio not exceeding 1:2 with respect to the metal chloride. DES System 2 consists of ferric chloride, with the FeCl_3_: [BMIM^+^] [Cl^−^] molar ratio not exceeding 1:1.5, and is the highest molar ratio (i.e., highest amount of metal chloride) among the DES systems examined in this study. DES System 3 is comprised of zinc chloride, with a ZnCl_2_: [BMIM^+^] [Cl^−^] ratio not exceeding 1:2. Finally, DES System 4 consists of aluminum chloride, with an AlCl_3_: [BMIM^+^] [Cl^−^] ratio not exceeding 1:4, and was the lowest ratio among those examined in this study.

To achieve high efficiency chromatographic columns for IGC, the stationary phase should be applied as a thin film on the inner wall of the fused silica capillary column. However, when metal‐containing DESs were deposited onto the untreated commercial fused silica capillary, droplets of DESs were observed to form on the inner wall, resulting in a chromatographic column with low separation efficiency. To increase the surface energy between the DES and capillary wall, silicon dioxide and sodium chloride were used to roughen the surface of the fused silica capillary, thereby increasing its surface energy. Figure S3 shows an optical microscope image of the coated stationary phase on untreated capillary in which droplets can be observed. After pretreating the capillary columns (see Figure S4), the wettability of the capillary wall for the DESs was improved, leading to higher column efficiencies.

### 3.1 Effect of Metal Chloride/[BMIM^+^] [Cl^−^] Molar Ratio on System Constants and Probe Molecule Retention

As anticipated, changes in the metal chloride/[BMIM^+^] [Cl^−^] (HBD/HBA) ratio produced alterations in the chromatographic retention characteristics of several probe molecules. For all DES systems discussed in this study, a consistent and smooth increase in the system constants was observed as the temperature was lowered as shown in Tables 3, 4, 5, 6. To examine the impact of the HBD:HBA molar ratio on solvation interactions, DES Systems 1, 2, 3, and 4 (see Table 1) were analyzed separately. The system constants for eutectic mixtures in each DES system indicate that they possess significant *n*–π and π–π interactions (*e*‐term), dipolarity (*s*‐term), hydrogen bond basicity (*a*‐term), and dispersive‐type interactions (*l*‐term) as all system constants representing those solvation interactions were higher than 0. The *n*–π and π–π interactions (*e*‐term) and dispersive‐type interactions (*l*‐term) remained relatively constant as the HBD:HBA molar ratio was changed across all four DES systems. However, the dipolarity/polarizability, hydrogen bond acidity, and hydrogen bond basicity of DES System 4 showed opposite tendencies to the other three DES systems based on the system constants when the HBD:HBA ratio was decreased.

**TABLE 2:** Retention factors of alcohols, nitroalkanes, haloalkanes, and aromatic compounds at 50°C as measured on stationary phases comprised of four eutectic mixtures.

Probe	DES System 1 (50°C)				DES System 2 (50°C)				DES System 3 (50°C)			DES System 4 (50°C)	
	FeCl_2_:[BMIM^+^] [Cl^−^]				FeCl_3_:[BMIM^+^] [Cl^−^]				ZnCl_2_:[BMIM^+^] [Cl^−^]			AlCl_3_:[BMIM^+^] [Cl^−^]	
	1:2	1:2.5	1:3	1:4	1:1.5	1:2	1:3	1:4	1:2	1:3	1:4	1:4	1:5
Methanol	3.26	4.52	5.21	6.27	1.26	3.54	5.57	7.18	1.50	2.35	2.98	11.01	6.79
Ethanol	3.21	4.37	4.95	5.95	1.52	3.85	5.70	7.24	1.58	2.28	2.92	8.62	5.89
1‐Propanol	6.01	8.17	9.17	11.00	3.15	7.74	11.04	13.70	2.48	4.14	5.36	14.93	10.68
2‐Propanol	2.38	3.19	3.55	4.20	1.34	3.07	4.29	5.20	0.97	1.66	2.14	5.13	4.07
1‐Butanol	11.87	15.90	17.47	21.16	6.61	15.84	21.89	26.47	4.01	7.56	9.72	21.22	21.93
2‐Butanol	4.25	5.58	6.11	7.27	2.58	5.68	7.69	9.26	1.54	2.85	3.64	8.63	6.55
1‐pentanol	22.24	30.12	32.35	39.37	13.94	31.80	42.18	49.93	8.44	13.73	18.48	39.71	37.98
Cyclopentanol	33.47	43.23	48.63	58.50	19.93	44.74	60.80	73.16	11.5	21.70	28.63	64.00	58.21
1‐Hexanol	40.47	54.33	57.77	69.37	27.62	61.37	78.66	91.64	14.72	25.07	32.43	89.20	71.00
Cyclohexanol	64.99	84.14	89.41	105.22	43.59	92.06	117.51	135.96	22.84	38.21	52.22	105.98	95.32
Nitromethane	3.19	3.52	3.65	3.96	3.21	3.57	3.97	4.31	2.70	3.22	3.43	3.87	4.38
1‐Nitropropane	4.93	5.20	4.97	5.07	7.65	7.25	6.55	6.17	3.64	4.02	4.28	4.08	4.53
1‐Chlorobutane	0.11	0.17	0.15	0.14	0.25	0.25	0.20	0.18	0.11	0.09	0.13	0.06	0.11
1‐Chlorohexane	0.57	0.63	0.58	0.51	1.21	1.13	0.88	0.66	0.46	0.43	0.45	0.37	0.42
1‐Chlorooctane	2.02	2.28	2.29	1.78	4.40	4.38	3.22	2.43	1.91	1.67	1.73	1.34	1.62
1‐Bromooctane	4.27	4.80	4.82	3.73	9.16	9.35	6.82	5.04	4.01	3.41	3.55	2.62	3.26
Acetophenone	92.69	92.82	85.52	84.98	174.30	141.87	115.31	101.16	70.39	70.42	72.37	57.29	68.22
Naphthalene	106.76	112.31	103.35	103.07	210.00	167.98	135.93	117.62	83.20	87.59	89.14	60.67	75.20

**TABLE 3:** System constants for the four eutectic mixtures with different FeCl_2_ to [BMIM^+^] [Cl^−^] molar ratios belonging to DES System 1.

DES	Temp. (°C)	System constants								
		*c*	*e*	*s*	*a*	*b*	*l*	*n* ^a^	*R^2^* ^b^	*F* ^c^
FeCl_2_:2 [BMIM^+^] [Cl^−^]	30	−3.24 (0.06)	0.29 (0.06)	2.46 (0.08)	5.25 (0.12)	0.38 (0.09)	0.62 (0.01)	40	0.99	1277
	40	−3.30 (0.06)	0.30 (0.06)	2.42 (0.08)	5.02 (0.12)	0.34 (0.10)	0.58 (0.01)	40	0.99	1132
	50	−3.32 (0.06)	0.29 (0.06)	2.35 (0.09)	4.83 (0.13)	0.27 (0.11)	0.54 (0.01)	40	0.99	869
FeCl_2_:2.5 [BMIM^+^] [Cl^−^]	30	−3.23 (0.06)	0.27 (0.06)	2.49 (0.08)	5.61 (0.13)	0.29 (0.10)	0.62 (0.01)	40	0.99	1118
	40	−3.28 (0.06)	0.27 (0.06)	2.44 (0.08)	5.41 (0.12)	0.25 (0.10)	0.58 (0.01)	40	0.99	1159
	50	−3.30 (0.06)	0.27 (0.06)	2.39 (0.08)	5.18 (0.12)	0.21 (0.10)	0.54 (0.01)	40	0.99	1060
FeCl_2_:3 [BMIM^+^] [Cl^−^]	30	−3.27 (0.06)	0.23 (0.07)	2.59 (0.09)	5.98 (0.13)	0.13 (0.11)	0.62 (0.01)	40	0.99	1085
	40	−3.30 (0.06)	0.24 (0.06)	2.51 (0.09)	5.74 (0.13)	0.12 (0.11)	0.58 (0.01)	40	0.99	1009
	50	−3.35 (0.06)	0.25 (0.06)	2.45 (0.09)	5.53 (0.13)	0.08 (0.10)	0.54 (0.01)	40	0.99	939
FeCl_2_:4 [BMIM^+^] [Cl^−^]	30	−3.32 (0.06)	0.27 (0.06)	2.67 (0.08)	6.34 (0.13)	0.12 (0.10)	0.60 (0.01)	40	0.99	1270
	40	−3.37 (0.06)	0.28 (0.06)	2.60 (0.08)	6.09 (0.13)	0.12 (0.10)	0.57 (0.01)	40	0.99	1180
	50	−3.39(0.06)	0.28 (0.06)	2.54 (0.08)	5.85 (0.12)	0.08 (0.10)	0.53 (0.01)	40	0.99	1183

^a^ *n*, number of probes subjected to MLRA.

^b^ *R* ^2^, correlation coefficient.

^c^ *F*, Fisher *F*‐statistic.

**TABLE 4:** System constants for the four eutectic mixtures belonging to DES System 2 with different FeCl_3_ to [BMIM^+^] [Cl^−^] molar ratios.

DES	Temp. (°C)	System constants								
		*c*	*e*	*s*	*a*	*b*	*l*	*n* ^a^	*R^2^* ^b^	*F* ^c^
FeCl_3_:1.5 [BMIM^+^] [Cl^−^]	30	−2.92 (0.06)	0.26 (0.06)	2.10 (0.08)	3.27 (0.12)	0.77 (0.10)	0.65 (0.01)	40	0.99	1017
	40	−2.98 (0.06)	0.25 (0.06)	2.06 (0.08)	3.09 (0.12)	0.73 (0.10)	0.61 (0.01)	40	0.99	1020
	50	−3.04 (0.06)	0.26 (0.06)	2.03 (0.08)	2.94 (0.12)	0.68 (0.10)	0.58 (0.01)	40	0.99	926
FeCl_3_:2 [BMIM^+^] [Cl^−^]	30	−3.01 (0.06)	0.20 (0.06)	2.21 (0.08)	4.70 (0.12)	0.56 (0.10)	0.65 (0.01)	40	0.99	1114
	40	−3.04 (0.05)	0.20 (0.05)	2.16 (0.07)	4.49 (0.11)	0.52 (0.09)	0.62 (0.01)	40	0.99	1233
	50	−3.09 (0.05)	0.20 (0.05)	2.12 (0.07)	4.35 (0.11)	0.44 (0.09)	0.58 (0.01)	40	0.99	1098
FeCl_3_:3 [BMIM^+^] [Cl^−^]	30	−3.10 (0.06)	0.20 (0.06)	2.41 (0.08)	5.59 (0.12)	0.34 (0.10)	0.63 (0.01)	40	0.99	1160
	40	−3.15 (0.06)	0.22 (0.06)	2.34 (0.08)	5.36 (0.12)	0.32 (0.09)	0.60 (0.01)	40	0.99	1206
	50	−3.19 (0.05)	0.22 (0.05)	2.29 (0.07)	5.14 (0.01)	0.31 (0.09)	0.56 (0.01)	40	0.99	1196
FeCl_3_:4 [BMIM^+^] [Cl^−^]	30	−3.22 (0.06)	0.23 (0.06)	2.55 (0.08)	6.20 (0.12)	0.26 (0.10)	0.62 (0.01)	40	0.99	1343
	40	−3.24 (0.06)	0.24 (0.06)	2.48 (0.08)	5.92 (0.12)	0.25 (0.10)	0.58 (0.01)	40	0.99	1217
	50	−3.28 (0.06)	0.23 (0.06)	2.42 (0.07)	5.70 (0.11)	0.20 (0.09)	0.55 (0.01)	40	0.99	1249

^a^ *n*, number of probes subjected to MLRA.

^b^ *R* ^2^, correlation coefficient.

^c^ *F*, Fisher *F*‐statistic.

**TABLE 5:** System constants for the three eutectic mixtures with varying ZnCl_2_ to [BMIM^+^] [Cl^−^] molar ratios belonging to DES System 3.

DES	Temp. (°C)	System constants								
		*c*	*e*	*s*	*a*	*b*	*l*	*n* ^a^	*R^2^* ^b^	*F* ^c^
ZnCl_2_:2 [BMIM^+^] [Cl^−^]	30	−3.25 (0.08)	0.27 (0.08)	2.55 (0.10)	4.56 (0.15)	0.14 (0.12)	0.59 (0.01)	40	0.99	680
	40	−3.29 (0.08)	0.28 (0.08)	2.49 (0.10)	4.31 (0.15)	0.12 (0.12)	0.55 (0.01)	40	0.99	629
	50	−3.33 (0.07)	0.28 (0.07)	2.43 (0.10)	4.07 (0.15)	0.10 (0.12)	0.52 (0.01)	40	0.99	634
ZnCl_2_:3 [BMIM^+^] [Cl^−^]	30	−3.41 (0.07)	0.26 (0.07)	2.72 (0.09)	5.61 (0.14)	0.00 (0.12)	0.61 (0.01)	40	0.99	902
	40	−3.38 (0.06)	0.29 (0.06)	2.61 (0.08)	5.24 (0.12)	0.02 (0.10)	0.56 (0.01)	40	0.99	1055
	50	−3.41 (0.07)	0.31 (0.07)	2.52 (0.09)	4.84 (0.13)	0.04 (0.11)	0.52 (0.01)	40	0.99	838
ZnCl_2_:4 [BMIM^+^] [Cl^−^]	30	−3.42 (0.08)	0.21 (0.08)	2.76 (0.10)	5.94 (0.16)	0.03 (0.13)	0.61 (0.01)	40	0.99	763
	40	−3.38 (0.06)	0.27 (0.06)	2.61 (0.08)	5.47 (0.12)	0.03 (0.10)	0.56 (0.01)	40	0.99	1009
	50	−3.35 (0.06)	0.29 (0.06)	2.50 (0.08)	5.08 (0.12)	0.00 (0.09)	0.52 (0.01)	40	0.99	1087

^a^ *n*, number of probes subjected to MLRA.

^b^ *R* ^2^, correlation coefficient.

^c^ *F*, Fisher *F*‐statistic.

**FIGURE 1:**
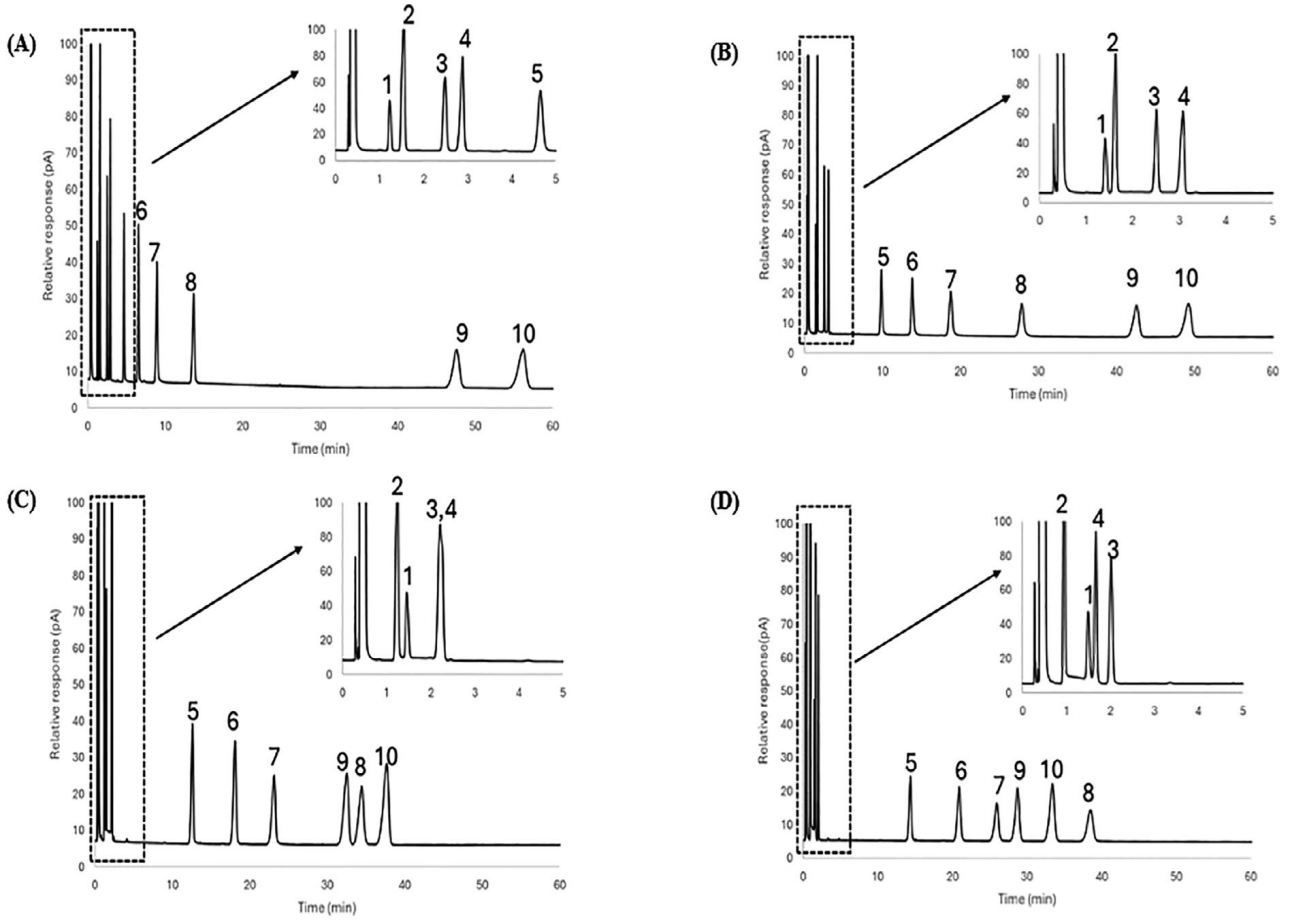
Chromatographic separation of alcohols, haloalkanes, nitroalkanes, and aromatic compounds showing the effect of varied metal chloride FeCl_3_ to [BMIM^+^] [Cl^−^] ratios for DES stationary phases at 50°C. (A) FeCl_3_:1.5 [BMIM^+^] [Cl^−^], (B) FeCl_3_:2 [BMIM^+^] [Cl^−^], (C) FeCl_3_:3 [BMIM^+^] [Cl^−^], (D) FeCl_3_:4 [BMIM^+^] [Cl^−^]. The inset within each chromatogram shows the first 5 min of the separation so that all chromatographic peaks can be observed. Analytes: 1—nitromethane; 2—1‐chlorooctane; 3—1‐nitropropane; 4—1‐bromooctane; 5—1‐pentanol; 6—cyclopentanol; 7—1‐hexanol; 8—cyclohexanol; 9—acetophenone; 10—naphthalene.

As the HBD/HBA ratio decreased from 1:2 to 1:4, the dipolarity (*s*‐term) at 50°C was observed to increase from (2.35 ± 0.09) to (2.54 ± 0.08) for DES System 1, (2.12 ± 0.07) to (2.42 ± 0.07) for DES System 2, and (2.43 ± 0.10) to (2.50 ± 0.08) for DES System 3. Among all of the system constants, the hydrogen bond basicity (*a*‐term) was observed to vary the most when the HBD:HBA ratio was changed. As the HBD:HBA ratio decreased from 1:2 to 1:4, the hydrogen bond basicity (*a*‐term) at 50°C increased notably from (4.83 ± 0.13) to (5.85 ± 0.12) for DES System 1, (4.35 ± 0.11) to (5.70 ± 0.11) for DES System 2, and (4.07 ± 0.15) to (5.08 ± 0.12) for DES System 3. These trends are further supported by increased chromatographic retention of alcohols. Although alcohols exhibit dipolar interactions and act as both HBDs and acceptors, their interactions with the stationary phase also serve as a reliable measure of hydrogen bond basicity, as the *a*‐term from the Abraham model appears to be the most sensitive to variations in Lewis acid content. The chromatograms for all HBD:HBA molar ratios are shown in Figure 1. Comparing the 1:1.5 to 1:4 molar ratios of DES System 2 in Table 2, the retention factors of 1‐hexanol and cyclohexanol at 50°C increased by 231.96% and 211.93%, respectively.

The hydrogen bond acidity (*b*‐term) also showed a noticeable change as the ratio of HBD:HBA varied. As the ratio decreased from 1:2 to 1:4, the hydrogen bond acidity (*b*‐term) at 50°C decreased from (0.27 ± 0.11) to (0.08 ± 0.10) for DES System 1, (0.44 ± 0.09) to (0.20 ± 0.09) for DES System 2, and (0.10 ± 0.12) to (0.00 ± 0.09) for DES System 3. These trends are supported by the decreased retention of aromatic compounds, as shown in Figure 1 for all HBD:HBA molar ratios of DES System 2. Comparing the 1:1.5 to 1:4 molar ratios of DES System 2 in Table 2, the retention factors of acetophenone and naphthalene at 50°C decreased by 42.02% and 44.02%, respectively.

**TABLE 6:** Two eutectic mixtures belonging to DES System 4 with different AlCl_3_ to [BMIM^+^] [Cl^−^] molar ratios and their corresponding system constants.

DES	Temp. (°C)	System constants								
		*c*	*e*	*s*	*a*	*b*	*l*	*n* ^a^	*R^2^* ^b^	*F* ^c^
AlCl_3_:4 [BMIM^+^] [Cl^−^]	30	−3.59 (0.08)	0.24 (0.08)	2.95 (0.10)	7.50 (0.16)	0.00 (0.13)	0.61 (0.01)	40	0.99	990
	40	−3.66 (0.08)	0.25 (0.08)	2.85 (0.10)	7.10 (0.16)	0.00 (0.13)	0.57 (0.01)	40	0.99	861
	50	−3.59 (0.09)	0.26 (0.10)	2.70 (0.13)	6.67 (0.19)	0.00 (0.15)	0.52 (0.02)	40	0.99	545
AlCl_3_:5 [BMIM^+^] [Cl^−^]	30	−3.45 (0.08)	0.23 (0.08)	2.87 (0.11)	7.00 (0.16)	0.00 (0.13)	0.61 (0.01)	40	0.99	852
	40	−3.50 (0.09)	0.25 (0.10)	2.78 (0.13)	6.70 (0.19)	0.00 (0.15)	0.57 (0.02)	40	0.99	906
	50	−3.48 (0.07)	0.22 (0.07)	2.70 (0.09)	6.27 (0.14)	0.00 (0.11)	0.53 (0.01)	40	0.99	882

^a^ *n*, number of probes subjected to MLRA.

^b^ *R* ^2^, correlation coefficient.

^c^ *F*, Fisher *F*‐statistic.

### 3.2 Comparison of System Constants and Analyte Retention for the FeCl_2_/[BMIM^+^] [Cl^−^] and FeCl_3_/[BMIM^+^] [Cl^−^] DESs

DES Systems 1 and 2 were selected to examine differences in system constants and probe molecule retention due to their shared use of iron chloride, with the key distinction being that one contains a divalent ion (Fe^2^⁺) and the other a trivalent ion (Fe^3^⁺). A comparison of retention factors for alcohols at 50°C (Table 2) demonstrates that they were retained more strongly in DES System 2 than in DES System 1, indicative of the higher hydrogen bond basicity in DES System 2 (FeCl_3_/[BMIM^+^] [Cl^−^]); however, the hydrogen bond basicity (*a*‐term) values from Tables 3 and 4 show that eutectic mixtures in DES System 1 possess higher hydrogen bond basicity than those in DES System 2. For instance, at 50°C, the hydrogen bond basicity (*a*‐term) for FeCl_2_/2 [BMIM⁺] [Cl^−^] was (4.83 ± 0.13), compared to (4.35 ± 0.11) for FeCl_3_/2 [BMIM⁺] [Cl^−^].

A comparison of retention factors for aromatic compounds at 50°C (see Table 2) shows that they are significantly more retained in DES System 2 than in DES System 1, indicative of a higher hydrogen bond acidity in DES System 2. This finding is further supported by the *b*‐term values from Tables 3 and 4 at 50°C in which the hydrogen bond acidity values are (0.27 ± 0.11) for the FeCl_2_:2 [BMIM^+^] [Cl^−^] DES and (0.44 ± 0.09) for the FeCl_3_:2 [BMIM^+^] [Cl^−^] DES.

**FIGURE 2:**
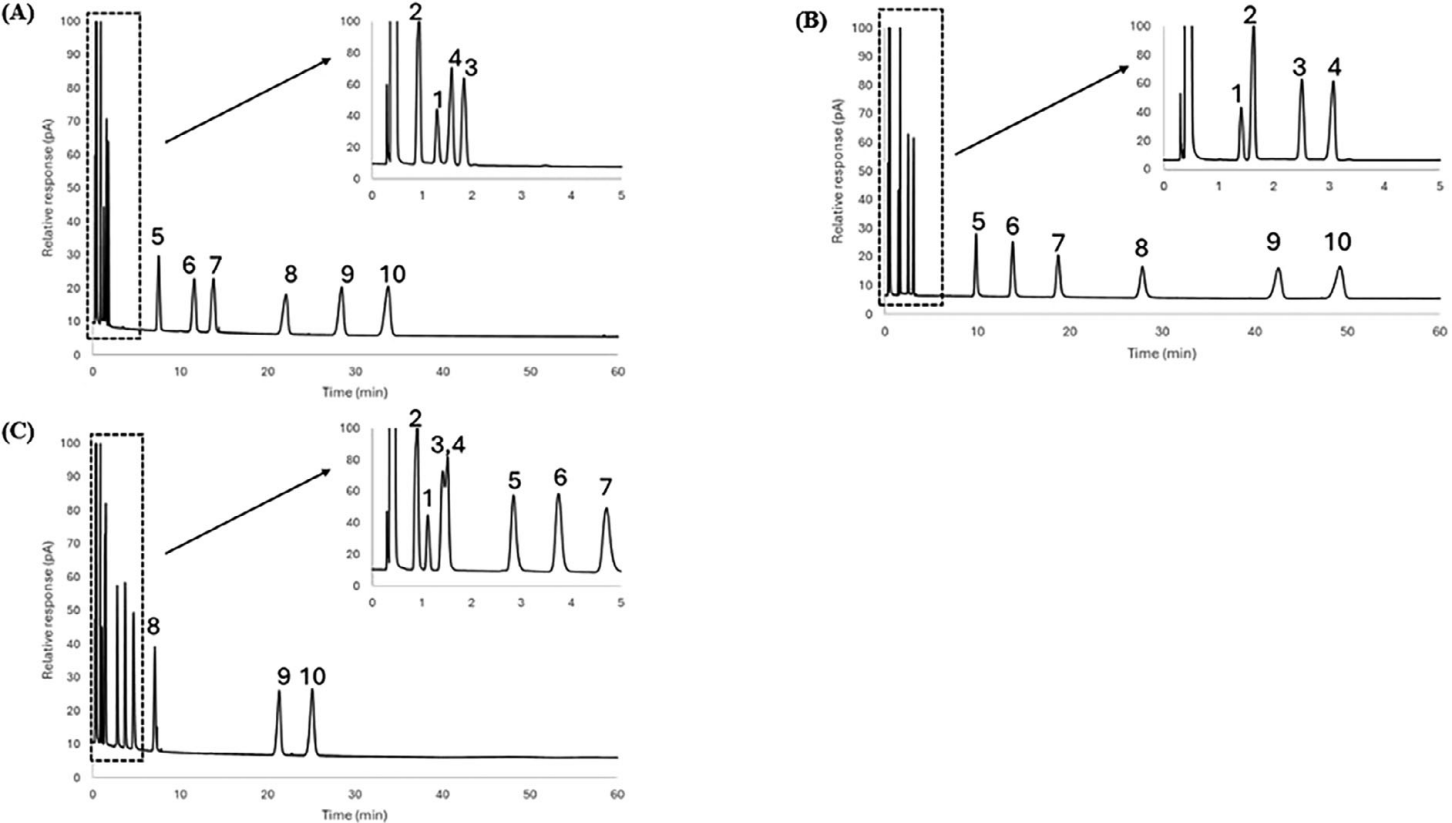
Comparison of chromatographic separation for (A) FeCl_2_:2 [BMIM^+^] [Cl^−^], (B) FeCl_3_:2 [BMIM^+^] [Cl^−^], (C) ZnCl_2_:2 [BMIM^+^] [Cl^−^] DES stationary phases at 50°C. The inset within each chromatogram shows the first 5 min of the separation so that all chromatographic peaks can be observed. Analytes: 1—nitromethane; 2—1‐chlorooctane; 3—1‐nitropropane; 4—1‐bromooctane; 5—1‐pentanol; 6—cyclopentanol; 7—1‐hexanol; 8—cyclohexanol; 9—acetophenone; 10—naphthalene.

**FIGURE 3:**
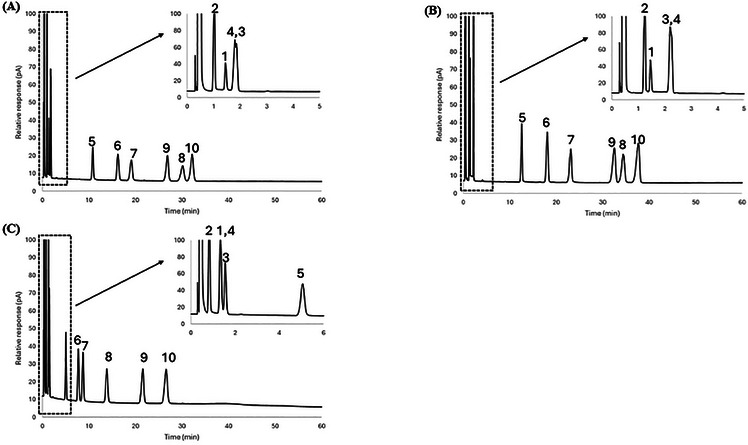
Chromatographic separation comparison for the stationary phases of (A) FeCl_2_:3 [BMIM^+^] [Cl^−^], (B) FeCl_3_:3 [BMIM^+^] [Cl^−^], and (C) ZnCl_2_:3 [BMIM^+^] [Cl^−^] DES at 50°C. Each chromatogram's inset displays the first 5 min of the separation so that all of the chromatographic peaks are visible. Analytes: 1—nitromethane; 2—1‐chlorooctane; 3—1‐nitropropane; 4—1‐bromooctane; 5—1‐pentanol; 6—cyclopentanol; 7—1‐hexanol; 8—cyclohexanol; 9—acetophenone; 10—naphthalene.

**FIGURE 4:**
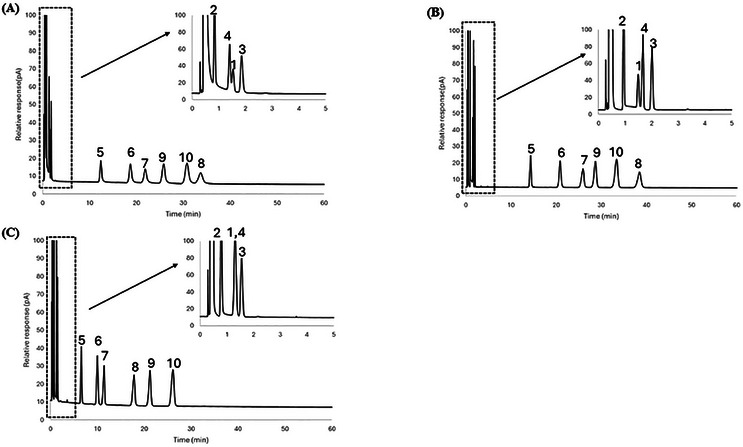
Comparative analysis of chromatographic separation for the stationary phases of (A) FeCl_2_:4 [BMIM^+^] [Cl^−^], (B) FeCl_3_:4 [BMIM^+^] [Cl^−^], and (C) ZnCl_2_:4 [BMIM^+^] [Cl^−^] DES at 50°C. To view all of the chromatographic peaks, the first 5 min of the separation are displayed in the inset of each chromatogram. Analytes: 1—nitromethane; 2—1‐chlorooctane; 3—1‐nitropropane; 4—1‐bromooctane; 5—1‐pentanol; 6—cyclopentanol; 7—1‐hexanol; 8—cyclohexanol; 9—acetophenone; 10—naphthalene.

A mixture of 10 probes was separated using the 1:2, 1:3, and 1:4 iron chloride: [BMIM^+^] [Cl^−^] ratios in DES Systems 1 and 2, and corresponding chromatograms are shown in Figures 2, 3, and 4A and Figures 2, 3, and 4B, respectively. Since both systems contain iron chloride as the metal chloride component, the elution order for the probe molecules on both eutectic mixtures was found to be similar across all metal chloride and [BMIM^+^] [Cl^−^] ratios. The 10 probes were generally well‐resolved at both iron chloride and [BMIM^+^] [Cl^−^] ratios, except for nitropropane (Probe 3) and 1‐bromooctane (Probe 4), which co‐eluted on both FeCl_2_/[BMIM^+^] [Cl^−^] and FeCl_3_/[BMIM^+^] [Cl^−^] eutectic mixtures at the 1:3 molar ratio composition.

### 3.3 Comparison of System Constants and Analyte Retention on FeCl_2_/[BMIM^+^] [Cl^−^] and ZnCl_2_/[BMIM^+^] [Cl^−^] DESs

DES Systems 1 and 3 were chosen to compare system constants and probe molecule retention as both systems feature divalent ions (Fe^2+^ or Zn^2+^) in their metal chloride components. A comparison of retention factors for alcohols at 50°C (see Table 2) shows that they were more strongly retained on stationary phases comprised of DES System 1 than in DES System 3, indicative of the higher hydrogen bond basicity for DESs in System 1. This observation is further reinforced by the hydrogen bond basicity (*a*‐term) values at 50°C from Tables 3 and 5, which were found to be (4.83 ± 0.13) for the FeCl_2_/2 [BMIM⁺] [Cl^−^] DES and (4.07 ± 0.15) for the ZnCl_2_/2 [BMIM⁺] [Cl^−^] DES, respectively.

A comparison of retention factors for aromatic compounds at 50°C (see Table 2) reveals that they were considerably more retained on DES stationary phases within DES System 1 than in DES System 3, highlighting the higher hydrogen bond acidity in DES System 1. This observation is further supported by the hydrogen bond acidity (*b*‐term) values reported in Tables 3 and 5; at 50°C, the hydrogen bond acidity for FeCl_2_/2 [BMIM⁺] [Cl^−^] is (0.27 ± 0.11), compared to (0.10 ± 0.12) for ZnCl_2_/2 [BMIM⁺] [Cl^−^].

To further illustrate the unique selectivity offered by the DESs examined in this study, a mixture of 10 probe molecules was subjected to separation using the 1:2, 1:3, and 1:4 metal chloride: [BMIM^+^] [Cl^−^] molar ratios for DES System 1 and 3. Chromatograms of these separations are shown in Figures 2, 3, and 4A, and Figures 2, 3, and 4C, respectively. The elution order of the probe molecules was found to be consistent across all molar ratios of ZnCl_2_ to [BMIM⁺] [Cl^−^]. However, in the FeCl_2_/[BMIM^+^] [Cl^−^] DES system at a 1:2 molar ratio, cyclohexanol (Probe 8) eluted faster than acetophenone (Probe 9) and naphthalene (Probe 10). As the molar ratio of FeCl_2_ to [BMIM⁺] [Cl^−^] decreased to 1:3, cyclohexanol was observed to retain longer than acetophenone, but eluted faster than naphthalene. When the ratio was further decreased to 1:4, cyclohexanol retained longer than both acetophenone and naphthalene. This behavior can be somewhat explained by the hydrogen bond acidity value at 50°C, which did not exhibit significant variation in DES System 3, with values ranging from (0.10 ± 0.12) to (0.00 ± 0.09) as the metal chloride to [BMIM^+^] [Cl^−^] molar ratio decreased from 1:2 to 1:4 (see Table 5). In contrast, for DES System 1, the hydrogen bond acidity value at 50°C decreased from (0.27 ± 0.11) to (0.08 ± 0.10) as the molar ratio of metal chloride to [BMIM^+^] [Cl^−^] decreased from 1:2 to 1:4 (see Table 3).

### 3.4 System Constants and Probe Molecule Retention for the AlCl_3_/[BMIM^+^] [Cl^−^] DES System

Since eutectic mixtures containing high molar ratios of AlCl_3_ and [BMIM^+^] [Cl^−^] remained solids at room temperature, AlCl_3_/4 [BMIM^+^] [Cl^−^] and AlCl_3_/5 [BMIM^+^] [Cl^−^] mixtures were prepared and were observed to be liquids at room temperature. Metal chlorides are considered as Lewis acids [41], and trends of the hydrogen bond basicity (*a*‐term) and hydrogen bond acidity (*b*‐term) values in DES System 1, 2, and 3 support this. It can be observed that as the ratio of metal chlorides to [BMIM^+^] [Cl^−^] decreases, the hydrogen bond basicity value increases while the hydrogen bond acidity value decreases (see Tables 3, 4, 5). However, for the AlCl_3_/[BMIM^+^] [Cl^−^] DES system, trends of hydrogen bond basicity and the hydrogen bond acidity were found to be opposite to those observed in DES Systems 1, 2, and 3. As the molar ratio of AlCl_3_ to [BMIM^+^] [Cl^−^] decreased from 1:4 to 1:5, the hydrogen bond basicity at 50°C decreased from (6.67 ± 0.19) to (6.27 ± 0.14) (see Table 6), while the hydrogen bond acidity was near zero (see Table 6). These data suggest that as the molar ratio of AlCl_3_ to [BMIM^+^] [Cl^−^] decreases, the hydrogen bond basicity of the system is lowered, and the DES offers negligible hydrogen bond donating ability. These trends are reflected in the lower chromatographic retention of alcohols at 50°C, as shown by chromatograms for two AlCl_3_: [BMIM^+^] [Cl^−^] molar ratios in DES System 4. As the AlCl_3_ to [BMIM^+^] [Cl^−^] molar ratio is decreased from 1:4 to 1:5 (see Table 2), retention factors for 1‐hexanol and cyclohexanol were observed to decrease by 20.40% and 10.06%, respectively, at 50°C.

**FIGURE 5:**
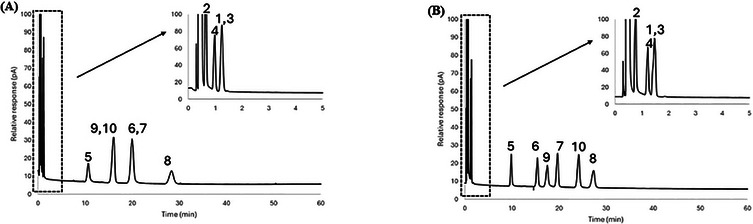
Alcohols, haloalkanes, nitroalkanes, and aromatic chemicals are separated chromatographically to demonstrate the impact of different metal AlCl_3_ on [BMIM^+^] [Cl^−^] molar ratios for DES stationary phases at 50°C: (A) AlCl_3_:4 [BMIM^+^] [Cl^−^], (B) AlCl_3_:5 [BMIM^+^] [Cl^−^]. Each chromatogram's inset displays the first 5 min of the separation so that all of the chromatographic peaks are visible. Analytes: 1—nitromethane; 2—1‐chlorooctane; 3—1‐nitropropane; 4—1‐bromooctane; 5—1‐pentanol; 6—cyclopentanol; 7—1‐hexanol; 8—cyclohexanol; 9—acetophenone; 10—naphthalene.

A mixture of 10 probe molecules were subjected to separation using the 1:4 metal chloride to [BMIM^+^] [Cl^−^] molar ratio across all DES systems (see Figures 4A–C and 5A). Generally, the 10 probes were well resolved in all the metal/[BMIM^+^] [Cl^−^] systems at a 1:4 molar ratio, except for the AlCl_3_:4 [BMIM^+^] [Cl^−^] DES. The AlCl_3_:4 [BMIM^+^] [Cl^−^] DES, identified as the most basic stationary phase in this study, exhibited the lowest separation efficiency for the 10 probes (see Figure 5A,B). When the AlCl_3_:4 [BMIM^+^] [Cl^−^] DES was used as a stationary phase, analyte pairs of nitromethane and 1‐nitropropane, acetophenone and naphthalene, and cyclopentanol and 1‐hexanol were observed to co‐elute. Figure S5 provides a summary of the type and magnitude of system constants for all DESs evaluated in this work. The figure serves as a guide for readers to aid in designing or customizing DESs with desired solvation characteristics.

## 4 Conclusions

Type I DESs that are formed by metal chlorides and imidazolium salts have gained popularity in separation science and metal processing applications due to their environmentally friendly nature and ease of preparation. The designer flexibility of Type I DESs allows for easy modification of their physical and chemical properties, as well as solvation interactions, by varying the Type of metal chloride and the molar ratio between the metal chloride and imidazolium salt in the eutectic mixture. For the DESs evaluated in this study, the hydrogen bond basicity, hydrogen bond acidity, and dipolarity/polarizability were the most affected when the metal chloride/imidazolium salt ratios were altered. Among all the probe molecules tested, alcohols and aromatic compounds exhibited the most significant changes in chromatographic retention as the metal chloride/imidazolium salt ratios were varied. Metal chlorides are Lewis acids, and the system constants of the hydrogen bond acidity in the first three systems of the study support this. In these systems, as the metal chloride/imidazolium salt ratio was increased, the hydrogen bond acidity of the eutectic system also increased. However, in the AlCl_3_/[BMIM^+^] [Cl^−^] system, the trend was reversed. While this trend does not disprove the fact that AlCl_3_ is a Lewis acid, it highlights that the Lewis acidity of different metal chlorides can vary significantly.

The results clearly demonstrate that chromatographic selectivity, resolution, and analyte elution order vary markedly across the different eutectic system studied, primarily as a result of distinct solvation interactions. Importantly, the solvation behavior of these systems was found to be highly sensitive to subtle changes in the metal chloride to imidazolium salt ratio. This pronounced tunability highlights the potential of DESs as highly customizable separation media, offering significant advantages for the rational design of DESs with desired solvation characteristics.

## Author Contributions

**Siyuan Liu**: experimental design, conceptualization, writing original draft, literature review. **Jared L. Anderson**: conceptualization, supervision, writing – review and editing, project administration, funding.

## Conflicts of Interest

The authors declare no conflicts of interest.

## Supporting information




**Supporting Information file 1**: jssc70231‐sup‐0001‐SupMat.pdf

## Data Availability

The data that support the findings of this study are available from the corresponding author upon reasonable request.
