# Supplementary material for: Measuring Solvation Interactions of Deep Eutectic Solvents Formed by Metal Chlorides and an Imidazolium Salt by Inverse Gas Chromatography
Source: J Sep Sci. 2025 Aug 5;48(8):e70231. doi: 10.1002/jssc.70231 (PMC12322802; doi:10.1002/jssc.70231)
Supplement: Supplementary file 1 — Supporting Information file 1: jssc70231‐sup‐0001‐SupMat.pdf [file JSSC-48-e70231-s001.pdf]

Supporting Information

Measuring solvation interactions of deep eutectic solvents formed by metal chlorides and an imidazolium salt by inverse gas chromatography

Siyuan Liu[1,2] and Jared L. Anderson[1,2,*]

[1]Ames National Laboratory, U.S. Department of Energy, Ames, Iowa 50011, USA

[2]Department of Chemistry, Iowa State University, Ames, Iowa 50011, USA

* Corresponding author. *E-mail address:* andersoj@iastate.edu (J.L. Anderson).

Table of Contents:

Figure S1……………………………………………………………..Page 2

Figure S2……………………………………………………………..Page 3

Figure S3……………………………………………………………..Page 4

Figure S4……………………………………………………………..Page 5

Figure S4……………………………………………………………..Page 6

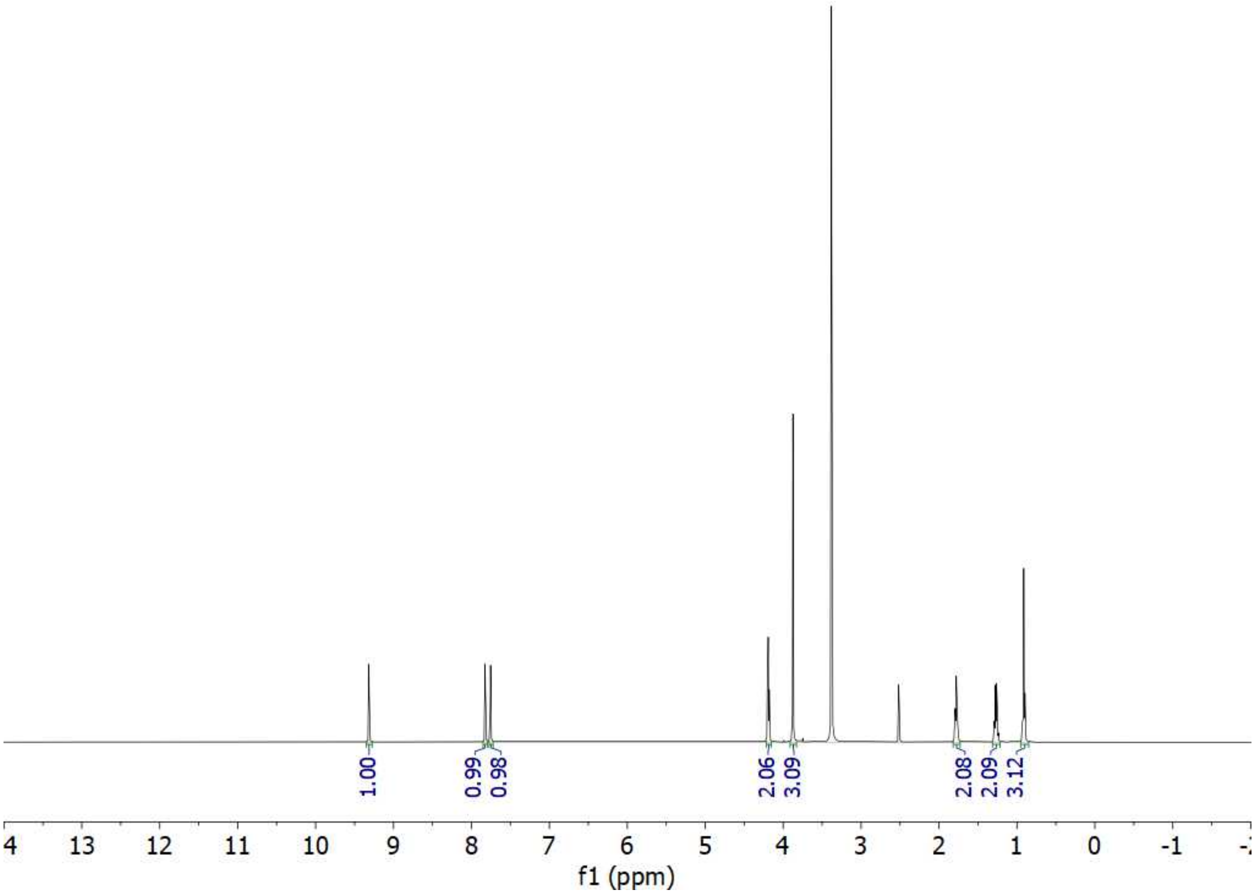

Figure S1. Proton NMR spectrum for 1-butyl-3-methylimidazolium chloride ([BMIM$^+$] [Cl$^-$]). ^{1}H NMR (600MHz: DMSO-d6; δ/ppm relative to TMS): 0.91 (3H, t, but-CH3), 1.26 (2H, m, CH2), 1.78 (2H, m, CH2), 3.87 (3H, s, NCH3), 4.19 (2H, t, NCH2), 7.75 (1H, s, NCH), 7.82 (1H, s, NCH), 9.32 (1H, s, NCHN).

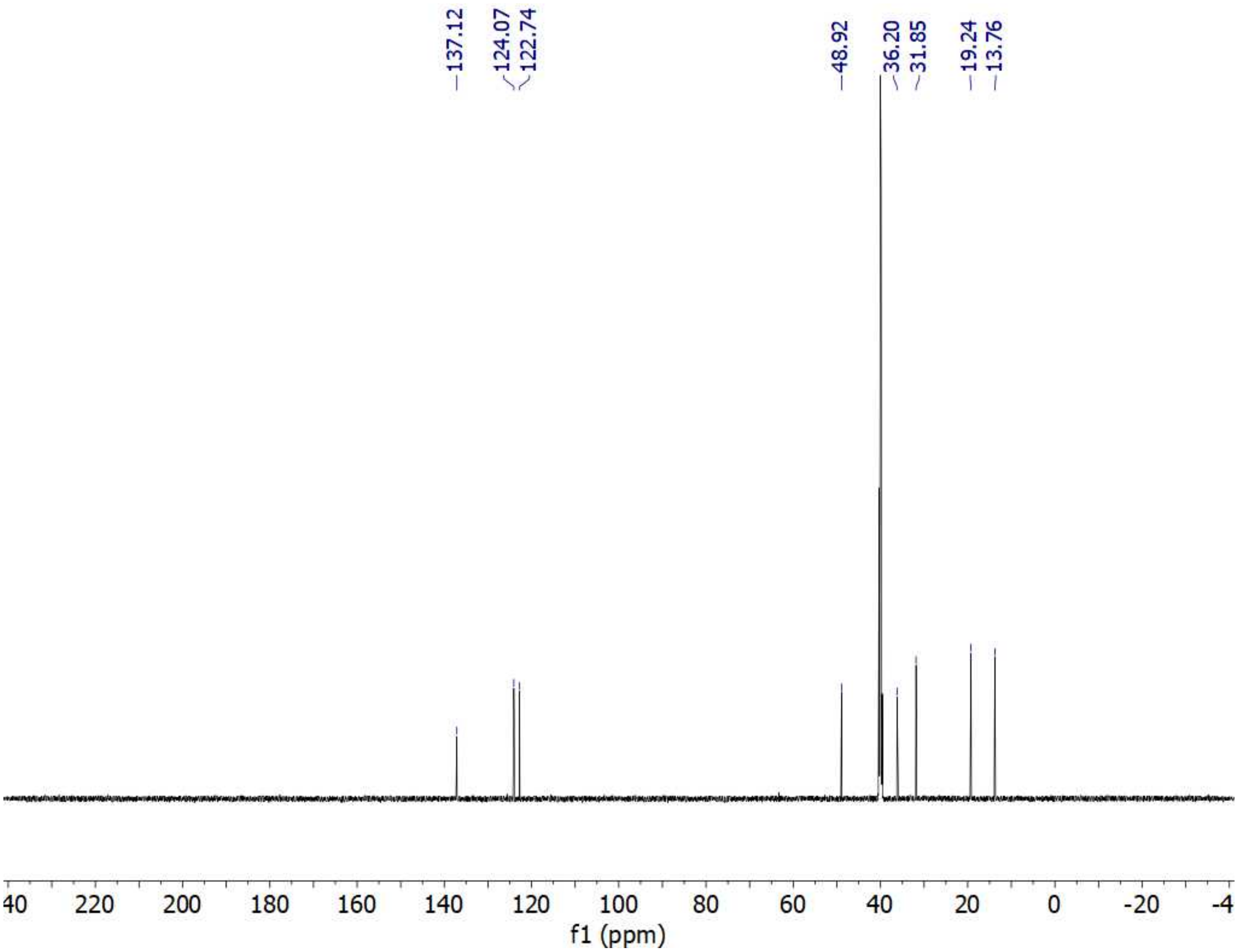

Figure S2. Carbon-13 NMR spectrum for 1-butyl-3-methylimidazolium chloride ([BMIM$^+$] [Cl$^-$]) IL. ^{13}C NMR (600MHz: DMSO-d6; δ/ppm relative to TMS): δ 13.76, 19.24, 31.85, 36.20, 48.92, 122.74, 124.07, 137.12.

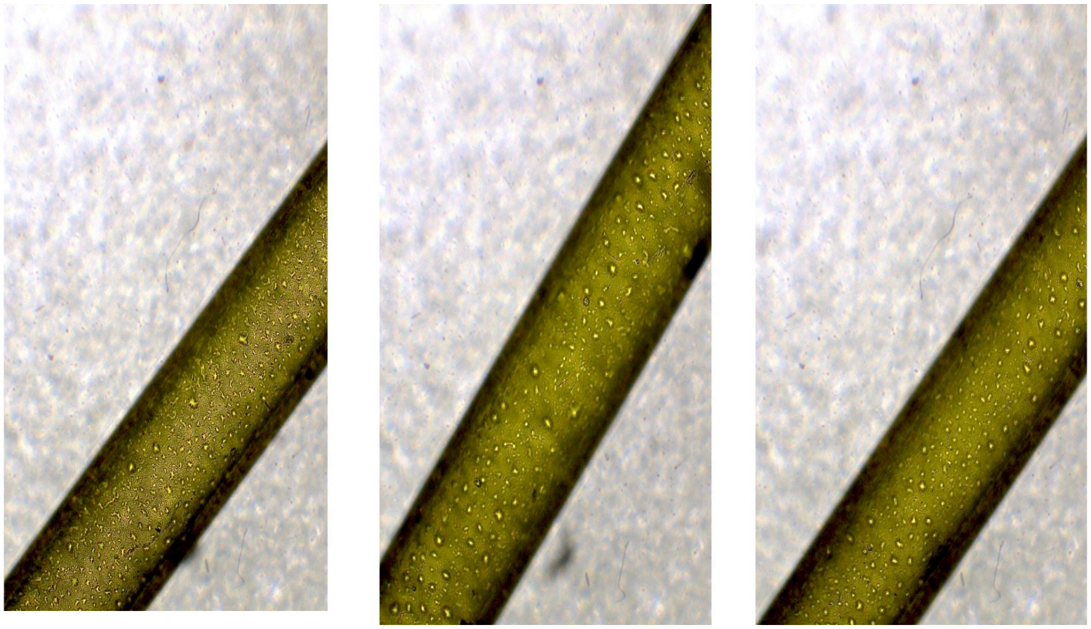

Figure S3. Optical microscopy images of DES system 1 coated on an untreated capillary column. The image on the far left is the beginning segment of the capillary column and the middle and right images are from the middle and end of the capillary (based on configuration of column during the static coating method), respectively. All three images show the DES stationary phase as droplets on the capillary column, which results in poor separation efficiency.

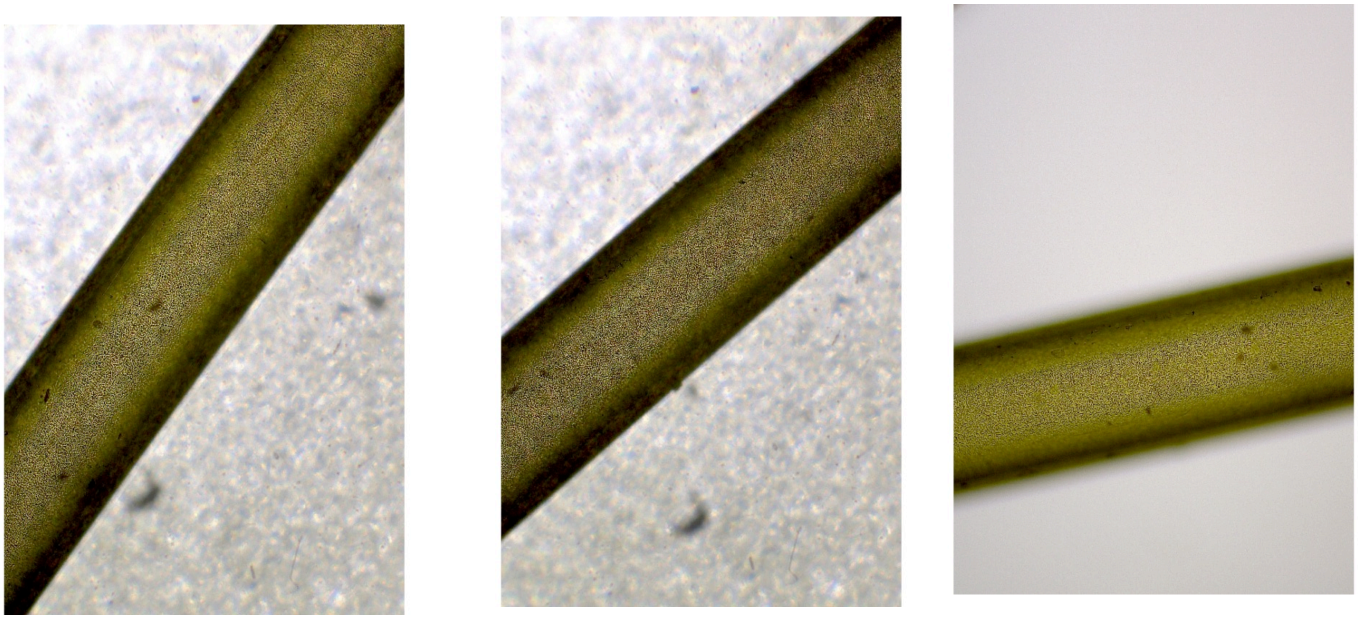

Figure S4. Optical microscopy images of DES system 1 coated on a capillary column subjected to the column pretreatment method described in this work. The image on the far left is the beginning segment of the capillary column and the middle and right images are from the middle and end of the capillary, respectively. Capillary columns coated with DESs after the pretreatment method produced high separation efficiencies due to a more homogenous film of the stationary phase on the inner capillary wall.

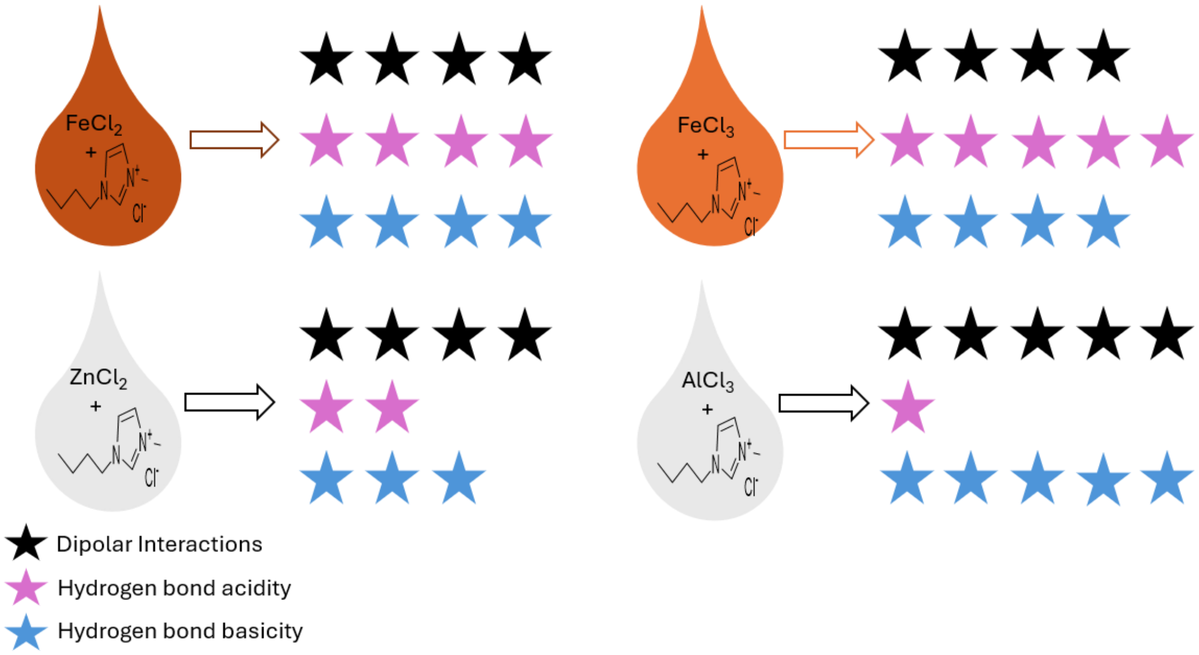

Figure S5. Graphic comparing the strength of individual solvation properties (dipolar interactions, hydrogen bond acidity, hydrogen bond basicity) for the metal containing DESs investigated in this study. A five-star rating denotes the highest value or magnitude for a particular solvation property.
